# Factors Affecting Contraceptive Use in Ethiopian: A Generalized Linear Mixed Effect Model

**DOI:** 10.4314/ejhs.v31i3.2

**Published:** 2021-05

**Authors:** Mulusew Admassu, Awoke Seyoum Tegegne

**Affiliations:** 1 John Snow Ink (JSI); 2 Statistics Department, Bahir Dar University

**Keywords:** Contraceptive use, predictor, Ethiopia, family planning, health facilities, Maternal Health

## Abstract

**Background:**

Ethiopia is the second most populous nations in Africa. Family planning is a viable solution to control such fast-growing population. This study aimed to assess the prevalence of contraceptive use and its predictors in Ethiopia.

**Methods:**

About 4,563 women were drawn randomly by Central Statistics Agency from its master sampling frame. The survey was conducted from January, 2014 to March, 2016 within six months' interval for the study period. The study was conducted using secondary data collected by PMA2020/Ethiopia project. Negative Binomial regression model was employed for data analysis. The model was selected using information criterion.

**Results:**

Predictors like easy access of health service, residence area, level of health institutions, regions, availability of community health volunteers, experience sharing, support from husbands, level of education and employment status of women as well as residence area significantly affected the performance of contraceptive use in Ethiopia. From the interaction effects of health centers with region and health post with number of opening days per a week were significant predictors of the contraceptive use.

**Conclusion:**

The performance of contraceptive use was different from one individual to another because of their experience sharing, support from their husbands, employment status and education level. A woman who got encouragement to use birth control from her husband had good performance to be effective for her contraceptive use. There should be an experience sharing/orientation, about use of birth control to protect women from unwanted pregnancy. Hence, rural women should get experience from urban women.

## Introduction

Globally, each year, nearly 350,000 women die while another 50 million suffer illness and disability from complications of pregnancy and child birth (1). Family planning (FP) is an important issue for many developing countries worldwide (2). Rapid population growth, which in many instances far outstrips of economic growth and environmental sustainability, is the reality in developing countries of Sub-Saharan Africa (3). Issues related to child bearing and birth control in the African continent, especially the sub-Saharan region, are of policy interest because of unusual increases in population growth that this area has experienced in the past decade (4). Sub-Sahara is among the major regions of the developing world that has not yet undergone a general decline in fertility (5). Health facilities related to family planning have a significant problem including staff refresher training, availability of running water, electricity, various equipment such as gloves and systems for sterilizing instruments are also problematic in several African countries (5). In developing countries millions of sexually active women of reproductive age (15 -49) want to avoid pregnancy and delay child bearing for at least two years or want to stop pregnancy and limit their family size but have unmet need for family planning(FP) (6).

Ethiopia being the second most populous nation in Africa, after Nigeria has been facing multitudes of challenges following rapid population growth including environmental degradation, chronic food insecurity, high maternal and child mortality rate. The total fertility rate of Ethiopia is about 4.6 children per woman with contraceptive prevalence rate (CPR) of 36% and an unmet need for FP of 22% for married women (6) and 24% for total women of age (15–9) years (7). If Ethiopia follows its current rate of growth, its population will be doubled in the next 30 years, hitting 210 million by 2060 (6). Most of the world's population growth in the next, 40–50 years is expected to come from Africa and Ethiopia will take a large share for this growth(8). This indicates that Ethiopia is among countries with the highest total fertility rates in the world (9).

At present, contraceptive methods in Ethiopia, which is free of cost is provided in both governmental and NGO health facilities including hospitals, clinics, health centers, and health posts (10). But, Ethiopia is among countries with low contraceptive prevalence rate, which is only 36% (6). This resulted in high total fertility rate and unwanted pregnancy which intern affects the maternal and child health status (11). Since, the country is a multiethnic and multi-cultural, it needs to investigate prevalence of contraceptive use and its predictors in the country. This is proposed by one of the previous studies, conducted about contraceptive use, in specific areas of country (12).

The Ministry of Health released Guidelines for Family Planning Services in Ethiopia to guide health providers and managers, as well as to expand and ensure quality family planning services in the country (13). The ministry designed new outlets for family planning services in the form of community-based distribution, social marketing, and work-based services. Moreover, in the last decade, integration and linkage between family planning services and HIV/AIDS care, along with maternal and other reproductive health services, have been emphasized in guidelines and strategic documents with the aim of enhancing family planning utilization (14).

Previous cross-sectional studies conducted in some specific areas in the country indicate that, reproductive age women in rural areas tend to be somewhat less ready for contraceptive use than those in urban areas. The reproductive age women in urban areas are well informed about contraceptive use as compared to those rural residences (15). However, this needs to check whether such variation is true across the country.

There is scarcity of longitudinal researches conducted recently about the prevalence of contraceptive use across the country at the same time using same data by same author. The gap was consider as potential study area for current investigation. As far as the authors' knowledge is concerned, in Ethiopia, there is scarcity of advanced and well-developed longitudinal research across the country, to assess covariates of service delivering for contraceptive use and fitting as a model. Therefore, the objective of current study was to investigate the prevalence of contraceptive use and its predictors in Ethiopian.

## Materials and Methods

**Study area and population**: The study was conducted in Ethiopia, across the country.

**Study design**: The data used under current investigation consists of secondary data and a retrospective longitudinal study design was employed. The sample for PMA2020/Ethiopia, used a two- stage cluster design with community and individual level.

**Source of data**: A secondary data obtained at Performance Monitoring and Accountability 2020 (PMA2020) project was used for current investigation. During data collection, the quality and collection procedure of data was facilitated by Johns Hopkins University, Bill & Melinda Gates Institute for Population and Reproductive Health from America, collaborating with Addis Ababa University School of Public Health, Central Statistical Agency, Federal Ministry of Health and Ethiopian Public Health Association. The survey was conducted from January, 2014 to March, 2016 within six months' interval for the study period on the selected health facilities across the country.

**Sample Size and sampling procedures**: A sample of 220 enumeration areas (EAs) was drawn randomly by CSA from its master sampling frame. From the enumeration areas, 382 health facilities from non-governmental, public and private health facilities located in eleven regions in the country were included. The data collection was controlled by supervisors selected in public health facilities and enumerators for private/NGO/other health facilities. Hence, random samples of 4,563 reproductive age women were included in current investigation across the country. The samples were selected using stratified random sampling technique, considering their regions and health facilities as strata.

**Eligibility Criterion:** All the reproductive age women included in the study were based on the criteria of family planning service with respect to the national guide line, and so all are eligible for this study.

**Variables under study**: Variables considered in this study were also selected based on the criterion of the PMA2020/Ethiopia project which has been taken as a main indicator and some of them from literatures which had been conducted at the global level. Potential determinant factors expected to be correlated with family planning were selected by the project.

**Response variable**: The prevalence of contraceptive use in the country was considered as the response variable, which is count or discrete response.

**Independent variables**: The total number of contraceptive use in the country is expected to be affected by several factors. The predictor variables included in the current study were Regions **(**Tigray, Afar, Amhara, Oromia, Somali, Benishangul-Gumz, SNNP, Gambela, Harari, Addis Ababa, Dire Dawa), Residence area (urban, rural ), Easy access of health service provision( yes, no), Orientation about use of birth control (yes, no), Availability of volunteers on health service provision(yes, no), Support of husbands/male partner (yes, no). Education level of women (illiterate, literate) , employment status of women (house wife, others). Among the potential predictors, region was considered as highest level variable and level of education, residence area support from husbands and employment status were lowest level variables related to individuals.

**Assumption of Model selection for count response data**: The type of model we can use for any investigation basically depends on the nature of response variables(16). Two wellknown models for count response variables are Poisson and negative Binomial models. The assumption of use of Poisson is equality of mean and variance, whereas, if variance greater than mean, negative Binomial is used for such distribution(16). Since, variance was greater than mean for the outcome variable (number of contraceptive use), negative Binomial was in favor of Poisson and selected for data analysis in current investigation. Model selection was also conducted using Akakai information criterion (AIC) and Bayesian information criterion (BIC) and among the count response models, Negative Binomial model was selected as shown in results section (Table 3). Similarly, covariance structure was also compared using information criterion and among the different covariance structures, compound symmetry was selected for data analysis(17).

**Table 3:** Goodness–of-fit test statistic result

Distribution	Fit Statistics	Value
	-2 Log Likelihood	20585
	AIC	20593
	AICC	20593.03
	BIC	20608.78
Poisson	CAIC	20612.78
	HQIC	20599.26
	Pearson Chi Square	8434.35
	Pearson Chi Square / DF	5.52
Negative binomial	-2 Log Likelihood	**15086.75**
	AIC	**15096.75**
	AICC	**15096.78**
	BIC	**15116.47**
	CAIC	**15121.47**
	HQIC	**15104.57**
	Pearson Chi Square	**1101.09**
	Pearson Chi Square / DF	**0.72**

**Generalized Linear Mixed Effect Model (GLMM):** To compute the random and fixed effect components, generalized linear mixed effect model (GLMM), which includes both linear and non-linear models was employed in data analysis. In this model, the different levels of contraceptive use were considered. Hence, Contraceptive use at regional level, health institution level and at individual level were taken in to account in GLMM.GLMM has two components namely the fixed effect and random components, the random components belongs to variation at individual level and fixed part belong to the average contraceptive of the whole community.

**Parameter estimation**: The method of maximum likelihood estimation was used to estimate the parameters in the given model (16, 17).

**Measure of contraceptive use:** To measure performance of contraceptive use, first the performance at community level/ regional level was considered then performance of contraceptive use at health facilities was taken in to account. Finally, the close follow ups (performance) of individuals/women for taking pills/contraceptive was considered. Hence, multilevel data analysis was also employed for data analysis. Women are nested with in health institutes and health institutes are nested within regions. Therefore, three level data analysis were considered in contraceptive use performance in data analysis.

**Data analysis**: The data were analyzed using GLMM with negative Binomial count response model. The statistical software used in current investigation was SAS software, considering the selected compound symmetry covariance structure.

## Results

**Descriptive data analysis**: A total of 11 regions, 382 health facilities and 4,563 reproductive age women in the country were included to the current study. The baseline characteristics of study population are indicated in Table 1. The study was conducted at 28 (7.33%) clinics, 173(45.29%) health centers, 80(20.94%) health post, 74(19.37%) hospitals and 27(7.07%) pharmacies. From total of health facilities, 8.61%, 4.97%, 21.5%, 3.7 %, 1.6 %, 1.3%, 1.6%, 16.8%, 24.9 %, 2.6 % and 14.4% were taken from Addis Ababa, Afar, Amhara, Benishangul Gumuz, Dire Dawa, Gambela, Harar, Oromia, SNNP, Somalia and Tigray regions respectively (Table 1).

**Table 1:** Baseline data for variable included under investigation

Variable	Category	Health institutions		Individuals/Women	
			
		n	%	n	%
Regions	Addis Ababa(AA)	26	6.81	599	13.1
	Afar	19	4.97	340	7.5
	Amhara	82	21.47	739	16.2
	Benishangul	14	3.66	182	4.0
	Dire Dawa	6	1.57	189	4.1
	Gambela	5	1.31	203	4.4
	Harar	6	1.57	200	4.4
	Oromia	64	16.75	677	14.8
	SNNP	95	24.87	759	16.6
	Somali	10	2.62	179	3.9
	Tigray	55	14.40	496	10.9
Facility type(FT)	Clinic(CL)	28	7.33	543	11.9
	Health Center(HC)	173	45.29	683	15.0
	Health Post(HP)	80	20.94	602	13.2
	Hospital(HSP)	74	19.37	2548	55.8
	Pharmacy(PH)	27	7.07	187	4.1
Residence area	Urban	199	52.09	2956	64.8
	Rural	183	47.91	1607	35.2
Services obtained at	Public	327	85.60	3542	77.6
	Private	55	14.40	1021	22.4
Service Charge	Yes	55	14.40	1254	27.5
	No	327	85.60	3309	72.5
Stock out Experience	Yes	122	31.94	2345	51.4
	No	260	68.06	2218	48.6
Easy access of health service for	Yes			1725	37.8
	No			2838	62.2
Education level of women	illiterate			1333	29.2
	Literate			3230	70.8
Employment status of women	Gov't employed			1255	27.5
	Private			1353	29.7
	Household wife			1955	42.8

Among the total health facilities, 52.1 % of health facilities were located in urban area, 85.6 % of health facilities owned by government and free of service charge for family planning and 14.40% were private and in which all had a service charge. From all health facilities, 31.9 % experienced stock out (delivering alternative family planning methods) for at least one method within three months preceding the survey. Among the health facilities included under investigation, 35.6% of them had a community health volunteers for family planning.

Among the reproductive age women included in the investigation, 13.1%, 7.5%, 16.2%, 4%, 4.1%, 4.4%, 4.4%, 14.8%, 16.6%, 3.9% and 10.9% were taken respectively at AA, Afar, Amhara, Benishangul Gumuze, Dire Dawa, Gambela, Harar, Oromia, SNNP, Somalia and Tigray regions. Among the individual women included under investigation, 64.8% were taken at urban areas and 35.2% were taken in rural areas. The data in Table 1 also indicates that only 37.8% of the women had easy access of health services, 29.2% of the women were illiterate and 42.8% were household wife.

**Covariance structure and Model Selection:** Before inferential data analysis, covariance structures and appropriate model selection were conducted using information criterion as indicated in Table 2 and Table 3 respectively.

**Table 2:** Information criteria to select covariance structure

Criteria (smaller is better)	CS	UN	AR(1)	TOEP
AIC	20593.00	20596.31	20593.05	20599.00
AICC	20593.03	20596.71	20593.03	20599.07
BIC	20608.78	20663.38	20608.78	20626.62
CAIC	20612.78	20680.38	20612.78	20633.62
HQIC	20599.26	20622.92	20599.26	20609.96

In Table 2, it is indicated that compound symmetry had smaller results on AIC, BIC and for other criterion compared with the other variance covariance structures. After selection of a model, the goodness fit was tested for potential candidates using test criterion and indicated in Table 3. Table 3 indicates that out of the two count response variable models, negative Binomial had smaller Pearson Chi Square/ DF and it was selected for data analysis.

In Table 3, for the Poisson model, the Pearson Chi-square value divided by the degrees of freedom was 5.52 which was greater than 1 and for the negative Binomial model, the Pearson Chi-square value divided by degree of freedom was 0.72 which is less than 1 and also the result of AIC for negative Binomial distribution was 15096.75 which is less than the AIC of Poisson distribution (20593). Hence, negative Binomial model was in favor of Poisson model.

**Multi variable data analysis**: To conduct the multi variable mixed effect model, first type I analyses was conducted to identify significant variables with p-value ≤ 0.25 that would be included to multi-variable analysis. The multi variable data analysis is indicated in Table 4. From Table 4, there were two estimated variance components; these are the random effects variances and the residual variance. The random variance component indicates that, the total variability of contraceptive use between regions was estimated to be 1.1065, whereas, the total variability of contraceptive use between health facility was 0.2104. Therefore, the total individual variation for contraceptive use was estimated to be 0.2104 + 1.1065 = 1.169. The multilevel data analysis in current investigation indicates that, the proportion of total variability that is attributed to individuals/women was given by 0.2104/1.3169 which is equal to 16%, while the proportion of total variability attributed to the between region variations in their level of contraceptive use was 1.1065/1.3169 which is equal to 84%. Therefore, more variation was explained by the random effects of individuals served at different health institutions in different regions. To investigate the variation of contraceptive use and predictors for these variations, potential predictors are included and indicated in Table 4.

**Table 4:** Multi-variable data analysis for parameter estimation

Effect	Estimate	e^β^(OR)	SE	P>|t|
Intercept	3.8059	45.07	0.6960	<.0001*
Health Facilities (Ref=PH).				
CL	-0.07757	0.9254	0.9399	0.9343
HC	0.5339	1.7055	0.7816	0.4951
HP	-2.0323	0.1310	0.8212	0.0139*
HSP	0.7021	2.018	1.0202	0.4919
Region (Ref=Addis Ababa)				
Afar	-1.2086	0.2986	0.7073	0.0878
Amhara	-0.3522	0.7031	0.6198	0.5699
Benishangul Gumuze	-0.9453	0.3886	0.7714	0.2206
Dire Dawa	0.3640	1.4391	0.8461	0.6671
Gambela	-0.4828	0.6171	0.7802	0.5362
Harar	-1.3864	0.2500	0.7344	0.0593
Oromia	-1.6153	0.1988	0.7188	0.0248*
SNNP	-0.2230	0.8001	0.5437	0.6818
Somalia	-0.2607	0.07705	0.7189	0.7170
Tigray	-0.3991	0.6709	0.6146	0.5163
Volunteer availability (Ref=Yes).				
No	-0.3409	0.7111	0.1502	0.0234*
Easy access of health service (Ref = yes)				
No	- 0.8929	0.4110	0.3874	0.0213*
Residence area(Ref.=Urban				
Rural	-0.4532	0.6356	0.5437	0.0316*
Experience sharing/ orientation about use of birth controls (Ref.= Yes)				
No	-0.042	0.958	0.632	0.001 *
Support from male partner to use birth control (Ref.=Yes)				
No	-0.81	0.445	0.46	0.032 *
Employment status of woman (Ref.= House hold wife)				
Others (private or gov't employee	-0.053	0.948	0.635	0.023*
Service charge/fee(Ref.= yes)				
No	1.5624	4.7703	0.3621	0.0213*
Stock out experience (Ref.=yes)				
No	-1.5725	0.2075	0.7437	0.0347*
Level of education* employment status (Ref. =House hold wife)				
Others (private or gov't employee)	-2.1991	0.1109	0.7507	0.0035*
Residence area* support from husbands (Ref=No)				
Yes	1.8048	6.085	0.7542	0.0169*
Random Effects				
variance estimate	G11		0.2213	
	G22		0.2213	
	G33		0.2213	

* stands for statistics significant at 5% level of alpha

In Table 4, both main and interaction effects significantly affected the number of contraceptive use in the study area. From the main effects, the availability of volunteers in providing health service, easy access of health services, experience sharing/orientation of women by the health staff, Support from the male partner, education level of woman, service charge for contraceptive use and employment status of women had significant effect on contraceptive use.

Hence, the log of expected number of modern contraceptive use in Oromia region was 0.80 (e^−1.6153^) times expected number of contraceptive use in Addis Ababa (P-value=0.0248). Hence, the performance of contraceptive use at Addis Ababa was significantly greater than Oromia and this further indicates that region had significant effect for variation of contraceptive use in the country.

The log of expected number of modern contraceptive use for which no community health volunteers in providing health service was 0.71 (e^−0.3409^) times expected number of contraceptive use that has a community health volunteers in health service provision(p-value=0.0234).

The log of expected number of modern contraceptive use of reproductive age women who had no easy access of health service was 0.41 (e^−0.8929^) times expected number of modern contraceptive use who had easy access, given the other variables constant.

Level of education had also played significant role for the variation of contraceptive use in the country. Hence, the log of expected number of modern contraceptive use for illiterate reproductive age women was 0.95 times the log of expected number of contraceptive use of literate reproductive age women.

Similarly, the log of expected number of modern contraceptive use who had no experience sharing was 0.958 times that of contraceptive use who had experience sharing. The log of expected number of contraceptive use woman who had no support from her husband was 0.445 (e^−0.81^) times expected number of contraceptive use of woman who got support from her husband. The log of expected number of contraceptive use of private or government employed women was 0.948 (e^−0.058^) times that of house hold wife, given the variables constant.

**Interaction effects**: Among all interaction effects in current investigation, only statistically significant effects, for the variable of interest, were taken for efficient use of space and for the table to be manageable within one page. The following were significant interaction effects obtained in current investigation.

**Interaction effect between level of education and employment status of women**: The expected number of contraceptive use of private or government employed women whatever the level of education was 0.11 (e^−2.199^) times the expected number of contraceptive use of household wife. Hence, employment status had significant effect for contraceptive use of reproductive age woman irrespective their education.

**Interaction effect between residence area and support from the husband**: The expected number of contraceptive use for reproductive age women who got support from their husband was 6.085 (e^1.8048^) times that of reproductive age women who did not get support from their husband.

## Discussion

This study was aimed to investigate the prevalence of contraceptive use and its predictors using longitudinal data within four rounds. The number of contraceptive use' data was analyzed using exploratory data analysis followed by model based outputs. Moreover, an average number of modern contraceptive use increased over time.

The performance of contraceptive use where volunteer health service providers are there is better as compared to those areas where no volunteer health service providers which is consistent with one of the previous investigations(18). The potential reason for this may be those volunteers facilitate fast service to deliver birth control and deliver orientation for clients about the use of family planning(19).

A reproductive age women, who lived in urban areas are more likely in using birth control as compared to those who lived in rural areas which is similar result obtained in research conducted previously (18).

A reproductive age woman who live in AA, are more likely in using birth control as compared to the other regions. The potential reason for this variation may be easy access of health services, level of education and support of women from husbands (20). A reproductive age woman employed as house hold wife are less likely in using birth control as compared to others. The reason for may be house hold wife women can treat their child because of no need of birth leave (21).

An illiterate woman in the reproductive age is less likely in using family planning as compared to literate women. The possible reason for this might be that level of education/level of thinking of women further helps to think consequence of having many children compared to their income(22). The other potential reason for this variation may be the level of thinking of ethnic groups, wealth index and other factors (6, 7). Culture at community level has its own side effect for use of birth control of reproductive age women (23).

Level of education or orientation about use of family planning plays significant role in performance of contraceptive use across the reproductive age women. Experience sharing among communities and individuals help to learn good and effective experience one from another(12).

Women who got support from their husbands to use birth control may be encouraged to visit health facilities to bring pills as compared to those who did not get support from husbands(24). The level of education of husbands may have significant role for performance of contraceptive use, this needs further investigation(25).

A reproductive age woman whose residence area belongs to rural area is less likely in using birth control as compared to urban areas. The reason for this might be the exposure and experience of the rural community (26). This indicates that, close follow ups and experience sharing/ orientation about use FP between rural and urban communities and between individuals make effective and had good trend (27, 28).

As a conclusion, this study investigated the performance of contraceptive use at regional/community level, health facility level and at individual levels in Ethiopia, considering longitudinal secondary data.

The study found that among the predictors, access of health services, type of health facility, region, existence of community health volunteers, orientation/experience sharing about use of birth control, level of education significantly affected the average performance of contraceptive use of a reproductive age woman in the study area.

On the other hand, from the interaction effects, easy access of the health service with orientation about use of birth control, facility type with region, facility type with easy access of the service were identified as significant predictors for the variation of performance of modern contraceptive use. A reproductive age woman who got health services easily had better prevalence rate on the number of contraceptive use as compared to the others.

Generally, number of contraceptive use was affected by regions, health facilities, experience sharing status, existence of volunteers around health facilities and employment status and education level of women.

For the PMA2020 project to be successful, there should be continuous experience sharing of reproductive age women about use of family planning. Experience sharing about use and advantage of contraceptive methods should be conducted at least once per week in each health facility to protect women from unwanted pregnancy.

The study had been conducted throughout the country which is not given much attention and not conducted in previously and may implicitly important for policy makers and health professional to revise the existed policy in the country, based on the information obtained in current study. The predictors for the variation of contraceptive use among reproductive age women will help for policy implications.
